# Transition from static culture to stirred tank bioreactor for the allogeneic production of therapeutic discogenic cell spheres

**DOI:** 10.1186/s13287-021-02525-0

**Published:** 2021-08-12

**Authors:** Daniel Rodriguez-Granrose, Jeff Zurawski, Will Heaton, Terry Tandeski, Galina Dulatov, Angelica Adrian Highsmith, Mason Conen, Garrett Clark, Amanda Jones, Hannah Loftus, Cameron LeBaron, Erin Scull, Niloo Farhang, Isaac Erickson, Justin Bingham, Paula Decaria, Nephi Jones, Kevin T. Foley, Lara Silverman

**Affiliations:** 1 DiscGenics Inc, 5940 Harold Gatty Dr, Salt Lake City, UT 84116 USA; 2 Department of Biochemistry and Molecular Biology, University of Miami, Miami, FL USA; 3 Department of Neurosurgery, University of Tennessee Health Science Center, Memphis, TN USA; 4 Thermo Fisher Scientific Inc, Logan, UT USA; 5 Semmes-Murphey Clinic, Memphis, TN USA

**Keywords:** Bioprocess, Progenitor cells, Cell spheres, Scale-up, Cell therapy, Stirred tank bioreactor (STR)

## Abstract

**Background:**

Culturing cells as cell spheres results in a tissue-like environment that drives unique cell phenotypes, making it useful for generating cell populations intended for therapeutic use. Unfortunately, common methods that utilize static suspension culture have limited scalability, making commercialization of such cell therapies challenging. Our team is developing an allogeneic cell therapy for the treatment of lumbar disc degeneration comprised of discogenic cells, which are progenitor cells expanded from human nucleus pulposus cells that are grown in a sphere configuration.

**Methods:**

We evaluate sphere production in Erlenmeyer, horizontal axis wheel, stirred tank bioreactor, and rocking bag format. We then explore the use of ramped agitation profiles and computational fluid dynamics to overcome obstacles related to cell settling and the undesired impact of mechanical forces on cell characteristics. Finally, we grow discogenic cells in stirred tank reactors (STRs) and test outcomes in vitro (potency via aggrecan production and identity) and in vivo (rabbit model of disc degeneration).

**Results:**

Computation fluid dynamics were used to model hydrodynamic conditions in STR systems and develop statistically significant correlations to cell attributes including potency (measured by aggrecan production), cell doublings, cell settling, and sphere size. Subsequent model-based optimization and testing resulted in growth of cells with comparable attributes to the original static process, as measured using both in vitro and in vivo models. Maximum shear rate (1/s) was maintained between scales to demonstrate feasibility in a 50 L STR (200-fold scale-up).

**Conclusions:**

Transition of discogenic cell production from static culture to a stirred-tank bioreactor enables cell sphere production in a scalable format. This work shows significant progress towards establishing a large-scale bioprocess methodology for this novel cell therapy that can be used for other, similar cell therapies.

**Supplementary Information:**

The online version contains supplementary material available at 10.1186/s13287-021-02525-0.

## Background

Allogeneic cell-based therapies have the potential to treat many conditions impacting large numbers of patients, each using a single source of cells. While techniques to generate the cells vary, one approach is to use cell sphere-forming culture methods that result in a three-dimensional, tissue-like environment. Current methods for this type of culture are small in scale and rely on complex or labor-intensive strategies [1, 2]. As such, developing scalable manufacturing platforms for cell spheres is essential to ensuring successful translation of such cell-based therapies into a commercial setting. This development is especially important if the therapy is intended to address large patient populations, such as those experiencing disc degeneration in the spine.

Disc degeneration is a major cause of low back pain, which is a leading cause of disability worldwide [3]. Patients with symptomatic disc degeneration who fail standard non-operative management have few therapeutic options. Some undergo fusion surgery, which has mixed outcomes [4, 5]. Approaches such as small molecules and biomaterials have demonstrated lack of efficacy and safety issues in clinical trials. Accordingly, novel treatments are needed to address this unmet medical need [6–8]. Cell therapy is a promising approach for this condition, where a live cell population is injected into the painful disc to drive new matrix production and minimize pain through active secretion of chemokines, extracellular matrix and other factors [9]. While a few clinical trials have been performed with cell therapies in the disc, none have shown robust efficacy and therefore, further research is needed to identify a suitable cell type [9].

One potential source of cells is nucleus pulposus tissue, the central portion of the intervertebral disc, from human donors. However, the prospect of this cell type for therapeutic purposes has historically been limited by its poor scale-up potential and subsequent inability to meet the need for large numbers of doses to treat patients, coupled with a loss of potency during outgrowth. In previous studies, cells derived from nucleus pulposus tissue were shown not to grow well in vitro and exhibit minimal matrix production [10, 11]. Our group has developed a method to overcome these two issues through a unique multi-week growth process that includes specific media components and culture conditions, resulting in a homogeneous cell population that we call Discogenic Cells, which are distinct from native nucleus pulposus cells and also produce extracellular aggrecan [12]. In preclinical animal models of disc degeneration, the Discogenic Cells showed restoration of disc height and normalization of tissue architecture, which we hypothesize will provide a potential benefit to human patients experiencing disc degeneration [12, 13]. Two double-blinded clinical trials are currently ongoing to evaluate Discogenic Cells in humans to investigate the safety and preliminary efficacy of this allogeneic cell therapy in the United States (clinicaltrials.gov identifier NCT03347708) and Japan (clinicaltrials.gov identifier NCT03955315).

Discogenic Cells used in preclinical studies and the ongoing clinical trials were produced using a static suspension culture that was manipulated using manual processes. For harvest, pooling individual flasks was required to achieve the desired lot size. The flasks contained a viscous, methylcellulose-based scaffold in the media to prevent cell adhesion to the flask surface and promote sphere growth, which drives the unique phenotype of Discogenic Cells seen at the end of the culture process [12]. Some other regenerative cell therapies also grow cells in sphere configuration because the three-dimensional tissue mimics endogenous tissue more closely than attachment or single cell suspension environments [1, 2, 12, 14–17]. However, for all of these applications, large-scale production is hindered by limitations caused by magnetic levitation, scaffolds, or viscous carriers [1, 2].

In contrast, our group decided to explore other scalable modalities such as stirred tank bioreactors (STRs), flasks with internal waterwheels, Erlenmeyer shake flasks, and wave rocking bioreactors. These systems still maintain suspension of cell spheres through motion caused by rocking or internally moving parts, therefore eliminating scaffold-related complications. We tested each of these modalities before selecting the STR as a platform technology. We sought to maintain the attributes of cell growth, sphere size, extracellular matrix production (specifically, aggrecan production), and flow cytometry identity through the transition from static to STR culture. One issue specific to growing progenitor cells in STRs is the potential for undesired cell characteristics imparted by mechanical forces [18–21]. Therefore, we used Computation Fluid Dynamics (CFD) to understand and optimize the STR parameters in an effort to meet the original process attributes. Finally, we performed an exploratory 200-fold scale-up in STR for large-scale production of Discogenic Cells. Successful scale-up of manufacturing is a key step towards enabling large-scale production of a cell therapy that ultimately can address the unmet medical need around disc degeneration and low back pain. Further, the findings may enable other cell therapies produced by cell sphere formation to address therapeutic areas outside of orthopedics.

## Methods

### Selection of new culture modality

Intervertebral disc tissue was obtained from recently deceased donors with an approved protocol (DonorConnect). Cells were isolated from the disc tissue using NB5 collagenase (Nordmark, Germany) and the isolated cells were expanded in vented cap T150 attachment culture flasks (Corning) in the presence of DMEM/F12 (Corning), amphotericin B (Mediatech), gentamicin (Mediatech), and a proprietary cocktail of other media supplements and intended to drive cell phenotype and further promote growth. Following attachment culture, cells from various donors were trypsinized using TrypLE (Thermo Fisher Scientific) and grown in the following non-adherent culture conditions: static suspension CellSTACK flasks with an ultra-low attachment coating with a viscous methylcellulose scaffold, rocking non-stick bags, Erlenmeyer shake flasks, Waterwheel vessels containing internal horizontal axis wheels, and STRs (Table 1). The static suspension CellSTACK flasks were filled with a viscous scaffold which mimics the clinical production process and served as the control group. All attachment and suspension conditions are grown with 5% Fetal Bovine Serum (FBS Defined US, Hyclone).

**Table 1:** Investigated culture modalities

Vessel	Supplier	Suspension Method	Working Volume (mL)	pH & Dissolved Oxygen control
CellSTACK (Static suspension)	Corning	Methylcellulose Static	165	Passive
Xuri Cell Expansion System	Cytiva	Rocking Platform	495	Passive
Erlenmeyer Flask	Chemglass	Orbital Shaking	125	Passive
Waterwheel	PBS Biotech	Horizontal Axis Wheel	100	Passive
DASbox (STR)	Eppendorf	Vertical Axis Impeller	250	Active

Cells were grown in four modalities with passive control and one modality with active control. The first modality, and control condition, was static suspension culture in CellSTACKs with an ultra-low attachment coating (Corning) (*n* = 8). In this static culture, media included 0.75% methylcellulose (Benecel A4M, Ashland) to provide a scaffold, prevent cell adhesion and promote sphere growth [12]. In the remaining modalities, cells were suspended with fluid movement in methylcellulose-free media. The second modality was 500 ml non-baffled, glass Erlenmeyer flasks with vented caps (Chemglass, *n* = 2 at 100 and 130 revolutions per minute (RPM)). Flasks were siliconized (Sigmacote siliconizing reagent, Sigma-Aldrich) prior to use in order to mitigate cell adhesion to vessel walls. The third modality was a vented cap waterwheel (PBS 0.1MAG, PBS Biotech, *n* = 2). The fourth modality was a Wave bioreactor (Xuri Cell Expansion System, Cytiva, *n* = 2). The reactor was rocked at a 4-degree rocking angle at 4 full oscillations per min. For each passively controlled modality, temperature was maintained at 37 °C. Dissolved oxygen and pH were maintained through a vented cap with 95% air and 5% CO_2_ using an incubator.

The fifth modality utilized active control, cells were grown in 0.25 L glass stirred tank bioreactor (0.25L DASbox Mini Reactor Systems, Eppendorf). STR vessels were siliconized with Sigmacote prior to use in order to mitigate cell adhesion to vessel walls. Six agitation speeds were tested using an 8-blade impeller (RPM = 75, 100, 125, 150, 175, 200). Dissolved oxygen and pH were actively controlled using a mixture of CO_2_, air, O_2_, and N_2_ gas overlay.

In each of the 5 modalities, cells were grown for comparable durations. Macroscopic images of the flasks/bioreactors and microscopic images of the cells/spheres were obtained at various magnifications immediately prior to harvest.

### Agitation Modeling Study

Twenty-two 0.25L STRs were run at a combination of static and ramped agitation speeds ranging from 75 to 225 RPM (Additional file 1). Cell doubling, sphere size, and extracellular aggrecan matrix production was measured for each condition. Then CFD was used to generate hydrodynamic models of fluid forces in the 0.25L STR (described below). These hydrodynamic models were compared to cell quality outputs using standard least squares regression. Finally, the STR parameters were modified to a new optimal set-point identified using the regression models of hydrodynamic forces and cell quality.

#### Cell doubling

At harvest, cells were dissociated out of spheres by applying a dissociation enzyme and placing the cells on a rocker until the spheres were dissociated. Then, a final cell number was obtained using a K2 automated cell counter (Nexcelom) to assess total growth kinetics. The cell doublings (doublings = 3.32 (log(harvest count) − log(inoculation count)) was reported as multiples compared to the doublings found in the static suspension culture.

#### Sphere size

To measure sphere size, 12 ml of culture suspension were harvested in 15 ml tubes (Falcon 15 mL Conical Centrifuge Tube, Thermo Fisher Scientific) by centrifugation for 10 min at 500×*g* (Legend XTR Centrifuge, Thermo Fisher Scientific). All supernatant except for 1 ml was aspirated and the cell pellet was resuspended in the remaining supernatant. Three samples per vessel were aliquoted onto a flat-bottom, 96-well plate, at 30 µl per well and imaged at 40 × magnification (CKX53 microscope and DP72 Camera, Olympus). Sphere size was then measured via image analysis using a custom ImageJ plugin generated in-house. [22]. For each photo, the custom plugin created an 8-bit grayscale image, removed outliers, filled holes, and used watershed to identify spheres and split up overlaid spheres. Finally, the plugin set scale and measurements to capture sphere characteristic data and adjusted for image magnification before the “Analyze Particles” function captured these measurements for all spheres between 1000 and 7500 pixels in size and 0.15–1.0 in circularity.

#### *Extracellular matrix protein *in vitro* culture assay*

Discogenic Cells were seeded in 96-well round bottom ultra-low attachment plates at 2.5 × 10^5^ cells/well in DMEM/F12 with 0.5% fetal bovine serum and 50 ug/mL gentamicin. Cell cultures were incubated at 37 °C with 5% CO_2_. Supernatant was removed from the culture for analysis by ELISA assays to determine the concentration of aggrecan (Aggrecan (PG) Human ELISA Kit, Thermo Fisher Scientific). An internal reference control was run with each assay to verify the assay performance.

#### Computation fluid dynamic modeling

Detailed models of two STR systems (0.25 L DASbox by Eppendorf and HyPerforma 5:1 50 L S.U.B. by Thermo Fisher Scientific) were created using DesignModeler, Meshing, Fluent & CFD Post (ANSYS 18.1, Ansys) The system was modeled using 7 agitation speeds which aim to capture the impeller’s RPM and hydrodynamic conditions scale. Average energy dissipation (m^2^/s^3^), power per unit volume (W/m^3^), maximum shear rate (1/s), average shear rate (1/s), volume average velocity (m/s) and tip speed (m/s) were calculated using a set of assumptions and equations found in the Additional file 1.

Cell doublings, sphere size, and aggrecan outputs from the twenty-two STR vessels were modeled using these CFD-derived hydrodynamic conditions and compared to the empirical findings using a standard least squares regression. CFD was also used to calculate the correlation between average eddy turbulence dissipation and distribution of the cells within each part of the reactor (cell volume fraction) assuming the spheres were the maximum observed sphere size from the STR runs.

### Comparison of static suspension and STR modalities

The STR parameters were updated to optimize doublings, sphere size, and aggrecan production values based on the agitation and CFD regression models. The static suspension and new STR culture processes were compared with cells derived from 8 distinct human donors cultured in parallel. A ramped agitation profile was used for the STR, where the agitation rate increased over time to account for the increase in sphere size. One replicate per static suspension condition and 3 replicates per 0.25 L STR were performed. The cells generated from each were compared for key attributes including cell doublings, sphere size, aggrecan expression, and flow cytometry identity. The bioactivity of the cells was also compared in an in vivo rabbit model of disc degeneration.

#### *Updated extracellular matrix protein *in vitro* culture assay*

For this comparison of static suspension and 0.25L STR studies, the assay was slightly modified to improve sensitivity. Discogenic Cells were seeded in 96-well v-bottom polypropylene plates at 2.5 × 10^5^ cells/well in DMEM high glucose with pyruvate supplemented with 1 × ITS + premix, 0.35 mM L-proline, 0.17 mM 2-phospho-L-ascorbic acid and 50 ug/mL gentamicin. Cell cultures were incubated at 37 °C with 5% CO_2_. Supernatant was removed from the culture for analysis by ELISA assays to determine the concentration of aggrecan (Aggrecan (PG) Human ELISA Kit, Thermo Fisher Scientific). An internal reference control was run with each assay to verify the assay performance.

#### Identity by flow cytometry

The cell identity was measured using flow cytometry with fluorochrome-conjugated mouse antihuman monoclonal antibodies, including appropriate isotype controls. The cells were incubated with antibodies at 4 °C for 30 to 60 min, in PBS with 0.5% human serum albumin, human Fc block and the following antibodies HLA-DR/DP/DQ, CD24, CD44, CD73, CD90, HLA-ABC, CD34, CD45, CD40, CD271, CD80, and CD86 (BD Biosciences, San Jose, CA, USA). Positive expression was assessed in the live cell population using 7-AAD (BD Biosciences) staining to exclude dead cells. The flow cytometry measurements were performed on a CytoFLEX Flow Cytometer (Beckman Coulter Life Sciences, Indianapolis, IN USA) and analyzed with FlowJo Software (BD Bioscience). No less than 10,000 events were collected for each analysis.

#### In vivo* evaluation of bioactivity*

For in vivo evaluation of bioactivity, female New Zealand White Rabbits (3–4 kg) were used under approval by a private Institutional Animal Care and Use Committee (IACUC). Fourteen rabbits were anesthetized and the lumbar region of the spine surgically exposed. Three lumbar discs (L3–L4, L4–L5, L5–L6) were injured via insertion of an 18-gauge needle 5 mm into the disc. L2–L3 was left undisturbed as a healthy control. Afterwards, muscle and skin were closed using sutures and the animals monitored during recovery. Rabbits were again prepared for surgery 2 weeks later, and the discs injected with 25 ml of 0 or 67,000 cells in vehicle from two different donors (*n* = 3–6/condition across 2 animals per condition) through a 27-gauge needle. The cell number was chosen based on previous studies showing bioactivity at this dosage [12]. Also, xenogeneic cells were utilized in order to avoid potential issues with comparability to the human product caused by known variability in native nucleus pulposus cell composition across species [23]. A sham procedure was also performed for comparison (*n* = 3). Half of the cells were generated in static culture containing methylcellulose and mixed with the traditional vehicle (1% sodium hyaluronate with Profreeze (Lonza Bioscience), dimethyl sulfoxide, saline and human serum albumin). The other half of the cells were generated in STR and mixed with an updated vehicle that excludes Profreeze.

X-ray images were obtained of the lumbar spine every 2 weeks. Measurements between 18 boney landmarks were taken in a blinded manner to calculate Disc Height Index (DHI) for the various conditions according to the method described in previous studies [12]. Also, animal body weight and behavior were noted for any abnormalities. Six weeks after dosing, the animals were sacrificed and the discs explanted, fixed, decalcified, sectioned through the center of the discs and stained with hematoxylin and eosin (H&E), Safranin O, and Picrosirius Red/Alcian Blue mixture. The slides were qualitatively evaluated by a blinded board-certified veterinary pathologist for the presence of abnormal tissue or inflammatory cells as well as any potential normalization of tissue architecture.

### Scale-up into pilot-scale STR

A CFD model of the HyPerforma 5:1 50 L S.U.B. (Thermo Fisher Scientific) was generated. RPM was scaled by maintaining maximum shear rate (s^−1^) between scales. Cells from a single donor were split and in the 0.25 L STR (DASbox, *n* = 2) and a 50 L pilot-scale commercial system (HyPerforma 5:1 50 L S.U.B. by Thermo Fisher Scientific, *n* = 1). The resulting Discogenic Cells were compared for doublings, sphere size, aggrecan and identity via flow cytometry using methods describes above.

### Statistical analysis

For each set of experimental conditions, averages and Standard Error (SE) were calculated. ANOVAs were conducted to compare conditions, where *p* < 0.05 was determined statistically significant. When comparing multiple groups, a standard least squares regression model was created and a post hoc least squares means differences student’s t test was conducted at alpha = 0.05 to compare experimental groups. All statistics were conducted in JMP Pro version 14.0.

## Results

### Selection of new culture modality

**Fig. 1:**
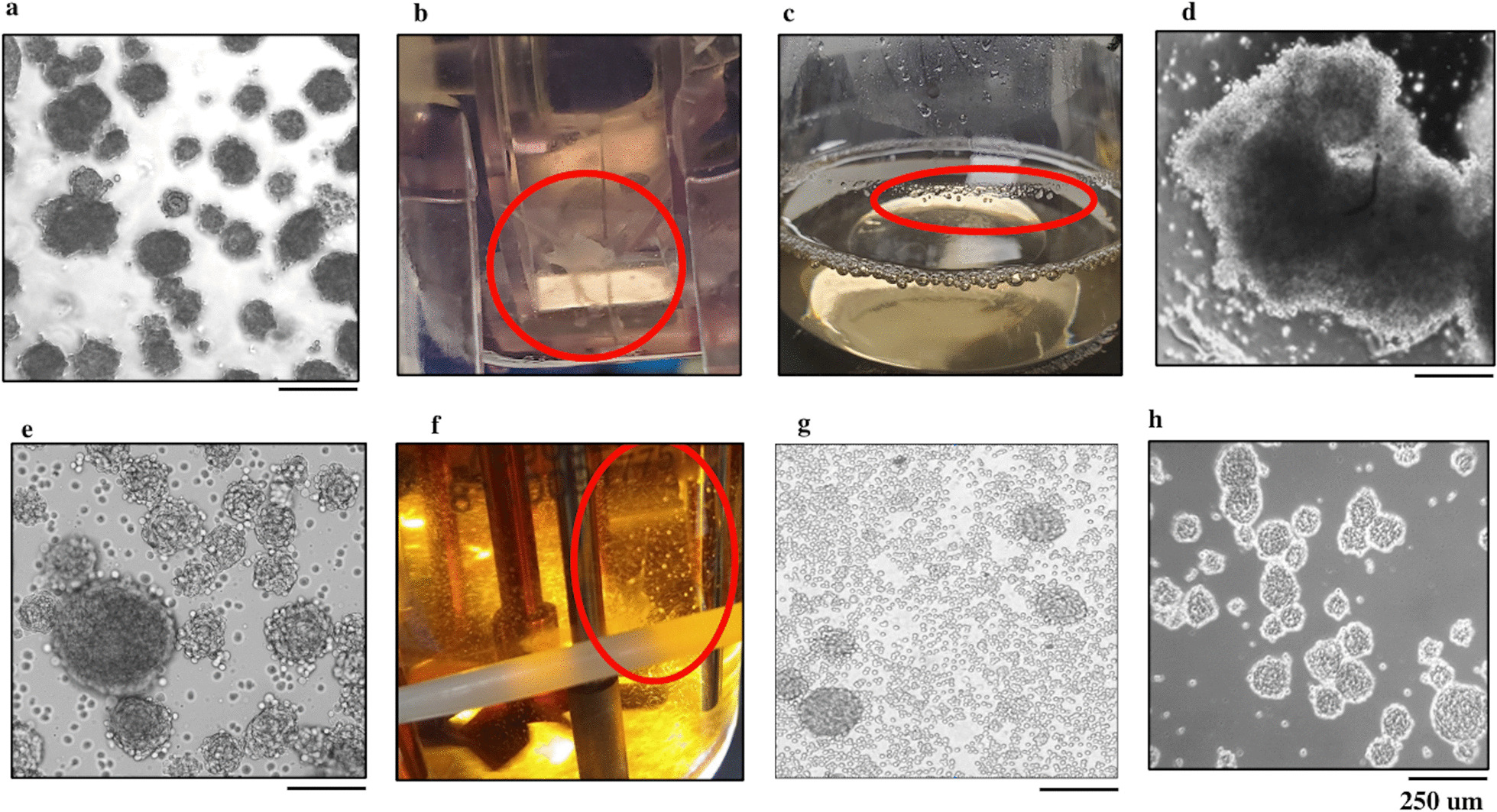
Investigated culture modalities. **a** Cells grown in a static suspension culture CellSTACK modality with methylcellulose exhibit the desired sphere phenotype. **b** Cells grown in waterwheel form into sheets rather than spheres. **c** Cells grown in Erlenmeyer flasks attach to vessel wall rather than in suspension. **d** Cells grown in Wave Bioreactor Bag form large rafts rather than spheres. **e** Cells form spheres when grown at low agitation in STR, however large spheres grow too large in size causing issues with oxygen transport and settling out of solution. **f** A large portion of cells grown at low agitation in STR attach to vessel surfaces impacting growth dynamics. **g** Cells grown at high agitation speeds in STR show some sphere growth but predominantly form single cells. **h** Using a ramped agitation profile with low initial RPM and high final RPM we are able to grow spheres in STR and limit cell attachment

Cells were grown in static suspension culture, wave reactors, waterwheels, Erlenmeyer shake flasks, and 0.25L STR. The cells grown in the static culture generated typical Discogenic Cell spheres (Fig. 1a). Cells grown in the waterwheels (3.87 doublings, SE 0.01) formed into long sheets of cells which trailed the wheel and built up in the vessel corners (Fig. 1b). Cells grown in Erlenmeyer shake flasks had similar doublings relative to the original static culture controls (100 RPM: 3.48 × doublings, SE 0.23; 130 RPM: 3.58 doublings, SE 0.18), but spheres were not generated and cells attached to the vessel walls and formed into clumps (Fig. 1c). Cell clumping was most egregious in the wave reactor where large rafts of cells formed (1.67 doublings, SE 1.46) (Fig. 1d).

In the 0.25L STR, cells grown at the low RPM setting formed spheres. However, spheres grew too large in size (Fig. 1e) causing issues with spheres settling out of solution (Fig. 1f); the medium and high RPM settings resulted in limited sphere formation (Fig. 1g). Doublings were consistently higher in the STR than in the original static suspension culture (75 RPM = 4.64 doublings, 100 RPM = 6.64 doublings, 125 RPM = 8.4 doublings, 150 RPM = 8.4 doublings, 175 RPM = 6.88 doublings, 200 RPM = 7.73 doublings). Because spheres formed at the low and medium RPM in the 0.25L STR, relative doublings were twice that of static suspension culture controls, and due to the potential for scalability, the STR modality was chosen for further optimization.

### Agitation modeling study

Next, an experiment employing twenty-two DASbox was run to evaluate different agitation strategies. No reactors run at a single RPM for the duration of culture enabled sphere growth while simultaneously limiting attachment to vessel walls. In contrast, when RPM was ramped over the course of the culture, we saw that small spheres successfully formed at the beginning of the run and then grow without settling. The ramped agitation allowed for sphere formation which visually resembled spheres from our static culture modality (Fig. 1h).

**Fig. 2:**
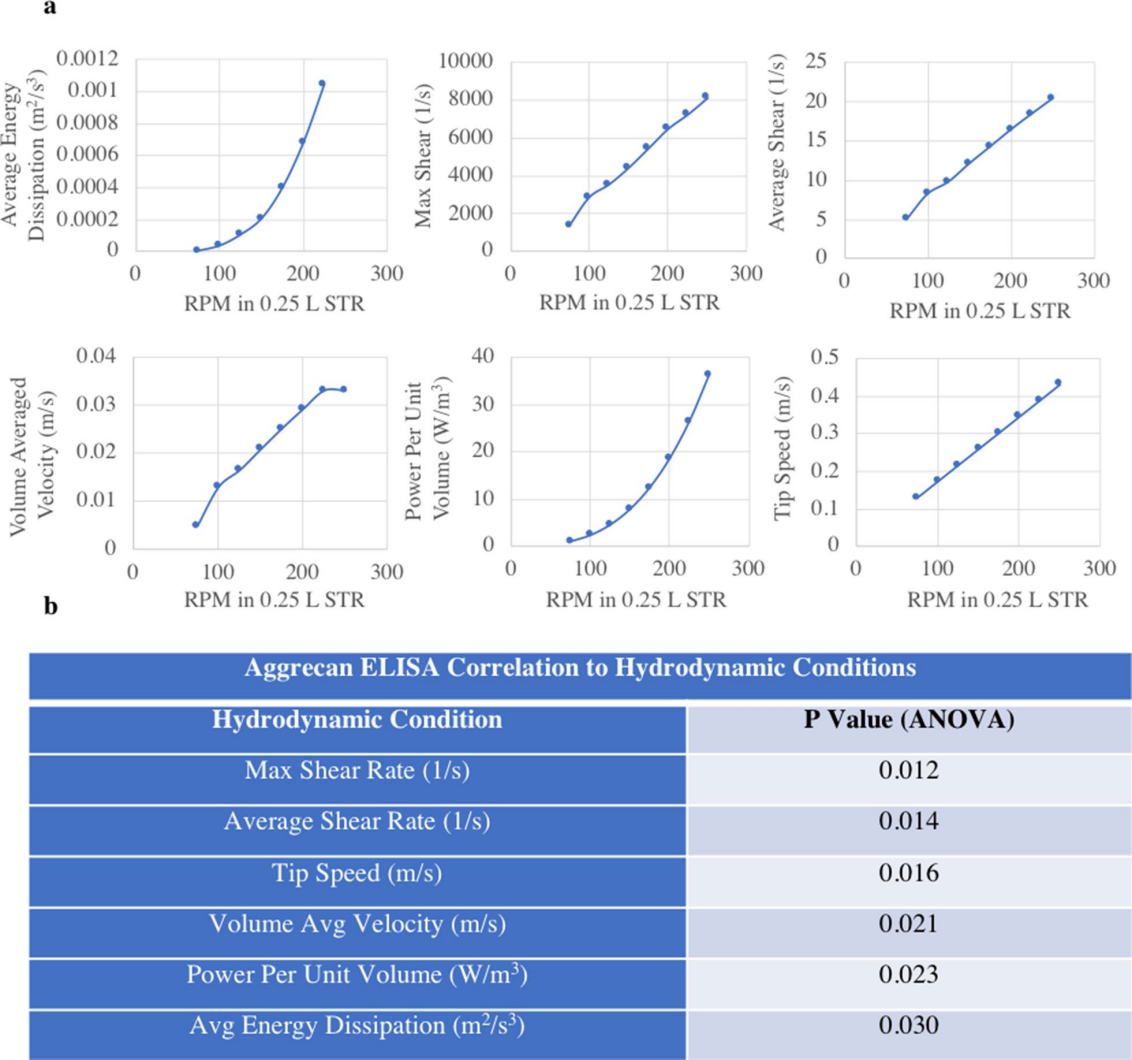
Variation in hydrodynamic conditions. **a** CFD models show that hydrodynamic conditions scale with different slopes and curvature based on RPM increase in STR, **b** by running STR at various conditions and then modelling aggrecan results using the various hydrodynamic slopes, we are able to see max shear rate (1/s) has the strongest correlation between our investigated hydrodynamic conditions and aggrecan expression (*p* = 0.012)

The data generated from these runs were used for CFD modeling of 8 different agitation rates. Hydrodynamic parameters were found to vary differently as a function of RPM (Fig. 2a). At the same scale but different RPM, average energy dissipation (m^2^/s^3^) and power per unit volume (W/m^3^) resulted in exponential curves whereas maximum shear rate (1/s), average shear rate (1/s), volume average velocity (m/s) and tip speed (m/s) had linear slopes. In each case the slope was different, indicating that the various hydrodynamic conditions do not scale equally based on RPM. By modeling aggrecan expression from the 22 DASbox reactors with the various hydrodynamic conditions, we found that maximum shear rate (1/s) had the highest correlation with aggrecan expression (*p* = 0.012) (Fig. 2b).

**Fig. 3:**
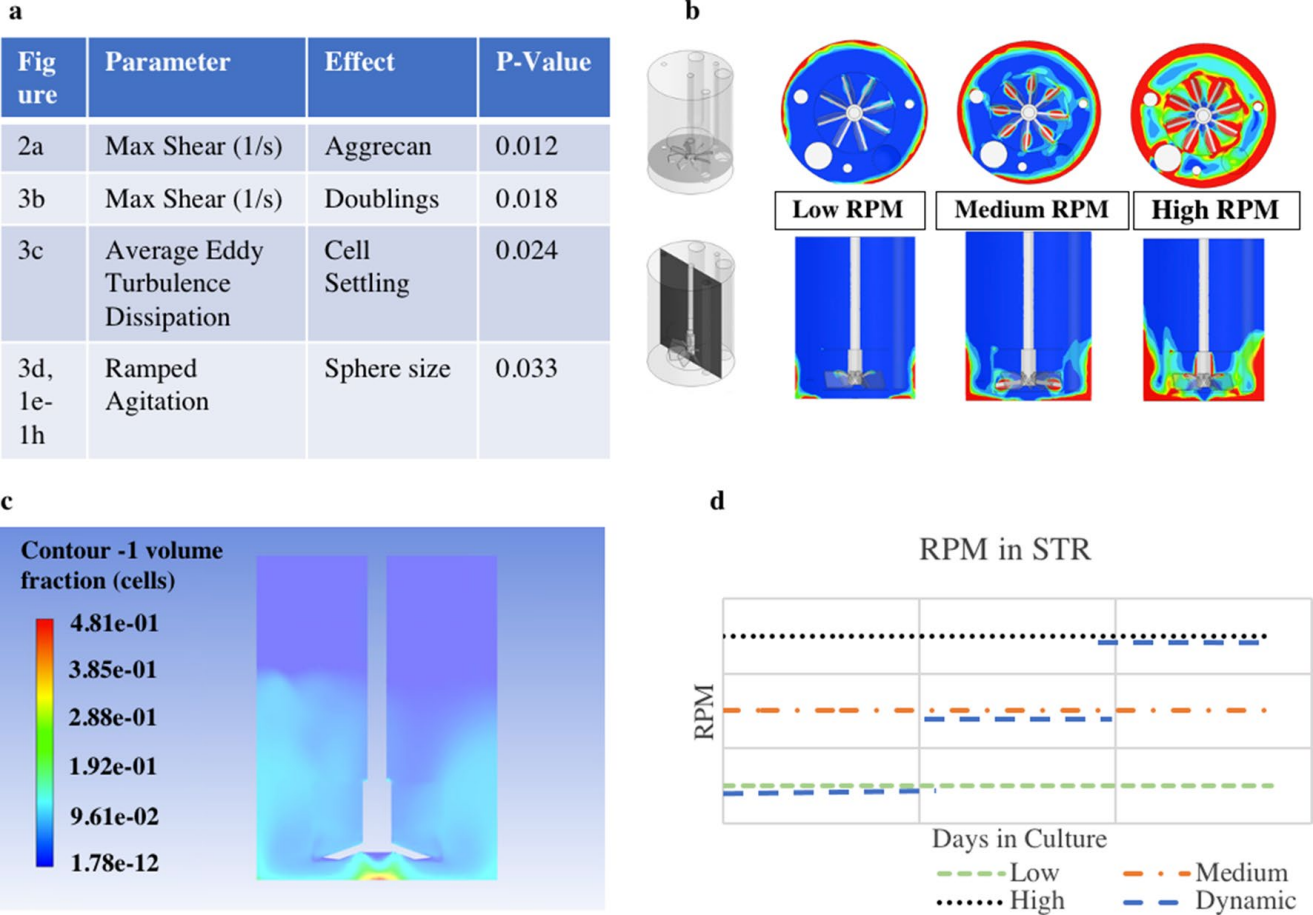
CFD models to enable sphere growth. **a** Correlation between hydrodynamic conditions and cell outputs include models of max shear rate and aggrecan (*p* = 0.012), average eddy turbulence dissipation and cell settling (*p* = 0.024), max shear rate and doublings (*p* = 0.018), and dynamic agitation with sphere size (*p* = 0.033). **b** Illustration of shifts in hydrodynamic environment as RPM shifts in dynamic culture conditions. By growing cells at various RPM and measuring outputs we can model effect of hydrodynamic conditions on cells. **c** Illustration of cell volume fraction calculated from average eddy turbulence dissipation at a single sphere size and RPM. Graphic shows simulated location of cells which can predict cell settling (other RPM, sphere sizes, and scales also simulated). **d** In order to minimize shear forces while also keeping spheres in suspension as they grow in size, we leverage CFD to develop dynamic agitation profiles

The CFD model of various agitation profiles showed us which metrics impact cell growth in STR (Fig. 3a). As previously described, max shear rate (1/s) impacted aggrecan production (*p* = 0.012), along with cell doublings (*p* = 0.018) (Fig. 3b). Also, cell settling was impacted by the cell volume fraction, meaning cell spheres would fall out of solution as predicted by average eddy turbulence dissipation (*p* = 0.024) (Fig. 3c). Ultimately, sphere size was impacted by the use of dynamic agitation, where the use of a ramping vs static agitation scheme significantly correlated with sphere sizes (*p* = 0.033) (Fig. 3d). With these findings, new set-points were identified for the DASbox which we predicted would produce Discogenic Cells with comparable properties to cells grown in static culture.

### Comparison of static suspension and STR modalities

**Fig. 4:**
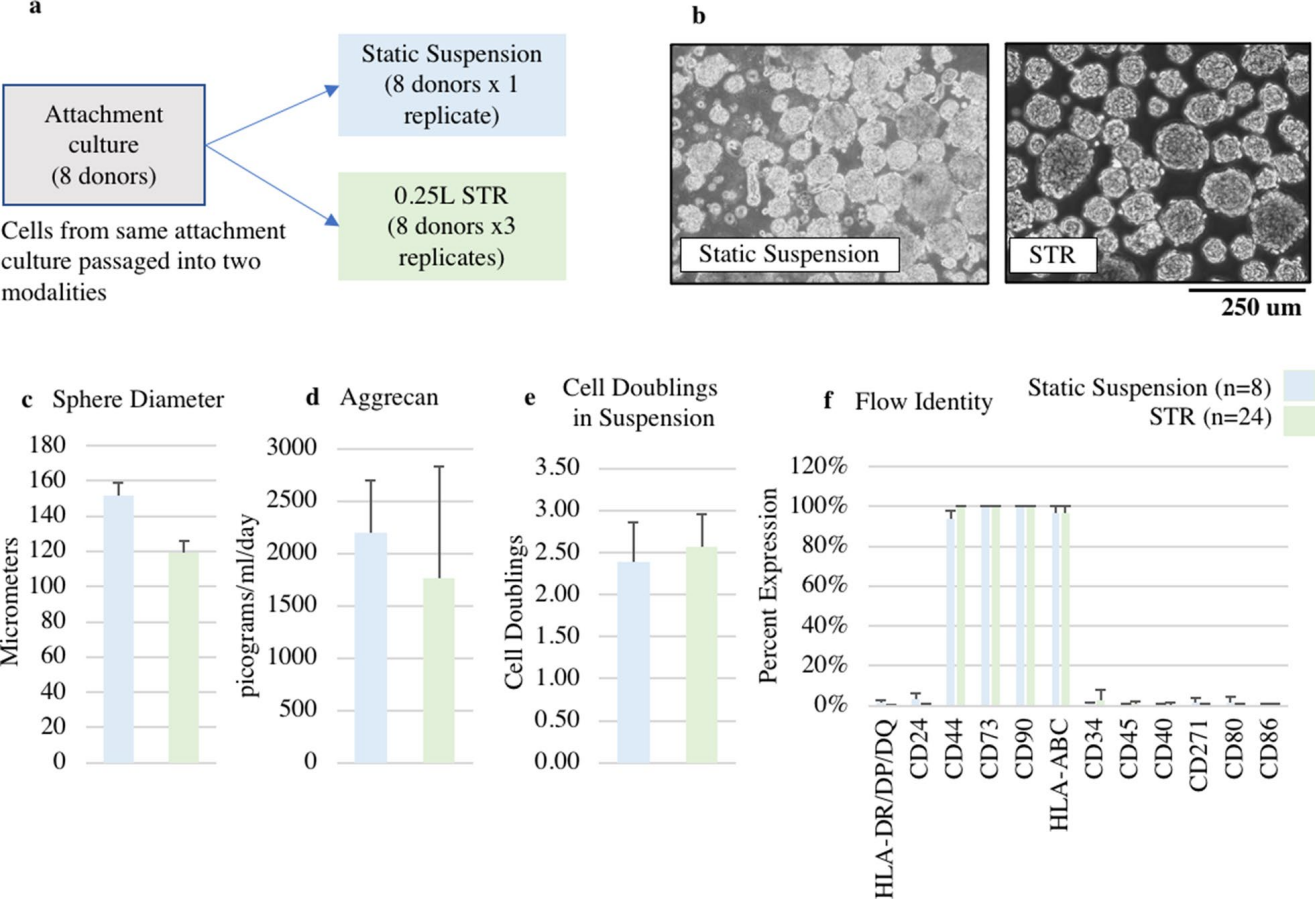
Cell characteristics in static suspension versus STR modality. **a** Diagram of split stream growth where 5 cell lines were grown in both 0.25 L STR and static suspension modalities. **b** Comparable sphere growth in static suspension culture and STR modalities when using ramped agitation. **c** Mean sphere size is slightly smaller in STR. **d** Aggrecan expression, and **e** doublings from day 2 to end of culture across static suspension culture and 0.25 L STR modalities are comparable. **f** Identity of cells as measured by flow cytometry is comparable between 0.25 L STR and static modalities

The agitation ramp profile that demonstrated the ability to generate spheres with minimal settling or sticking was carried forward and used for subsequent split stream growth between 0.25L STR and static suspension culture (Fig. 4a). In both modalities, all the cells formed the characteristic spheres (Fig. 4b). Average sphere size was slightly smaller in STR (120 µm, SE 6.3) compared to static controls (152 um, SE 6.8). (Fig. 4c). Aggrecan expression (Fig. 4d), doublings (Fig. 4e), and flow identity (Fig. 4f) did not exhibit statistically significant differences between the new STR process (Aggrecan Expressio*n* = 1760 picograms/ml day, SE = 1073; cell doublings = 2.57, SE = 0.4) and the original static suspension method (Aggrecan Expression = 2200 picograms/ml day, SE = 493; cell doublings = 2.38, SE = 0.5).

**Fig. 5:**
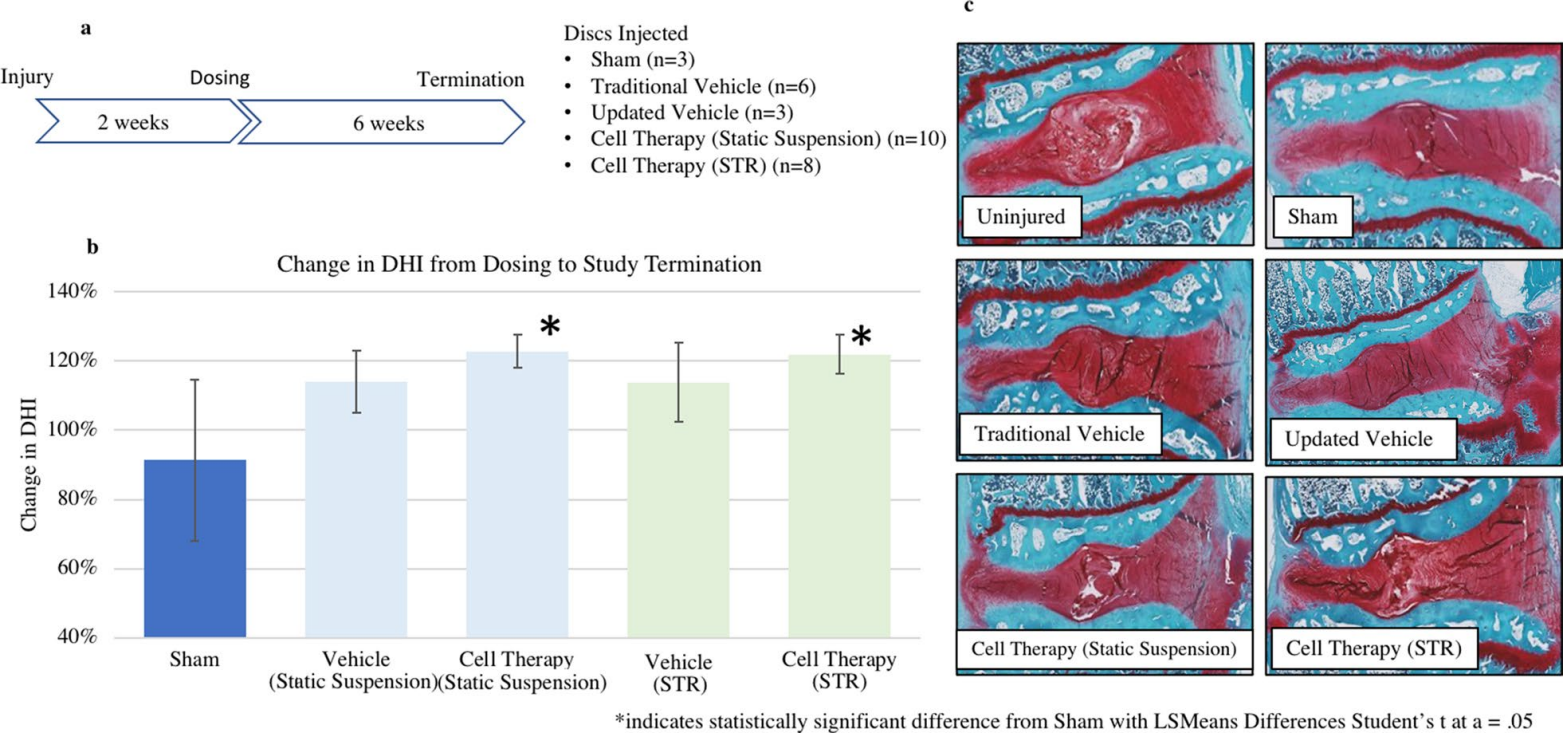
In vivo comparison of IDCT grown in static suspension versus STR culture. **a** The rabbit study design starts with disc injury, 2 weeks later the discs are dosed, 6 weeks after dosing the study is terminated. **b** Mean percent change in Disc Height Index (DHI) as measured from X-ray from dosing to termination is shown with standard error. After dosing, the disc height increased slightly in the sham group, more in the vehicle groups, and more substantially in the Cell Therapy groups. **c** Disc Histology shows height restoration and increase in hydration (white areas within center of red disc) for discs injected with Cell Therapy compared to vehicle or sham

Cells from both modalities were then tested in an in vivo rabbit model of disc degeneration. Following the initial injury, X-ray analysis showed that the disc height index decreased by 25–50%. After dosing (Fig. 5a), the disc height was restored slightly in the vehicle groups, and more substantially in the cell therapy groups (*p* = 0.019) (Fig. 5b). Cells grown using either process had statistically significant change in disc height index compared to the sham group after 6 weeks in vivo (*p* > 0.05) whereas vehicle did not. No host macrophage or t-cell infiltration was noted in histological evaluation.

Histological analysis by a pathologist identified abnormally high density and cellularity in the nucleus pulposus from discs that received sham or vehicle, hypothesized to be created by the loss of hydration caused by needle puncture injury and subsequent degeneration. In contrast, the discs injected with cell therapy had a more normal appearance in these parameters (Fig. 5c). Dorsal cartilage and osteophyte formation were noted but unassociated with treatment condition and are attributed to the injury model. No inflammation (as noted by the absence of inflammatory infiltrates) was noted that may have been caused by the cell therapy. Also, the new vehicle (which contains the original excipients minus one in different concentrations) did not result in any abnormal findings. Histologically, no abnormal tissue (fat, bone, etc.) was noted in the discs with cell therapy that could account for the structural increase in disc height measured via X-ray.

### Scale-up into 50 L STR

The cell volume fraction that was modeled indicated that a scale-up strategy based on tip speed (m/s) or average energy dissipation (m^2^/s^3^) would result in the majority of the cells settling out of solution. Also, scale-up maintaining average shear rate (1/s) would result in an extremely high RPM that would prevent sphere formation and promote cell death. Scale-up based on power per unit volume (W/m^3^), maximum shear rate (1/s), and volume average velocity (m/s) all should limit cell settling while allowing sphere formation. Of these hydrodynamic conditions, maximum shear rate (1/s) was chosen to scale into the 50 L because it had the strongest correlation with aggrecan expression.

**Fig. 6.**
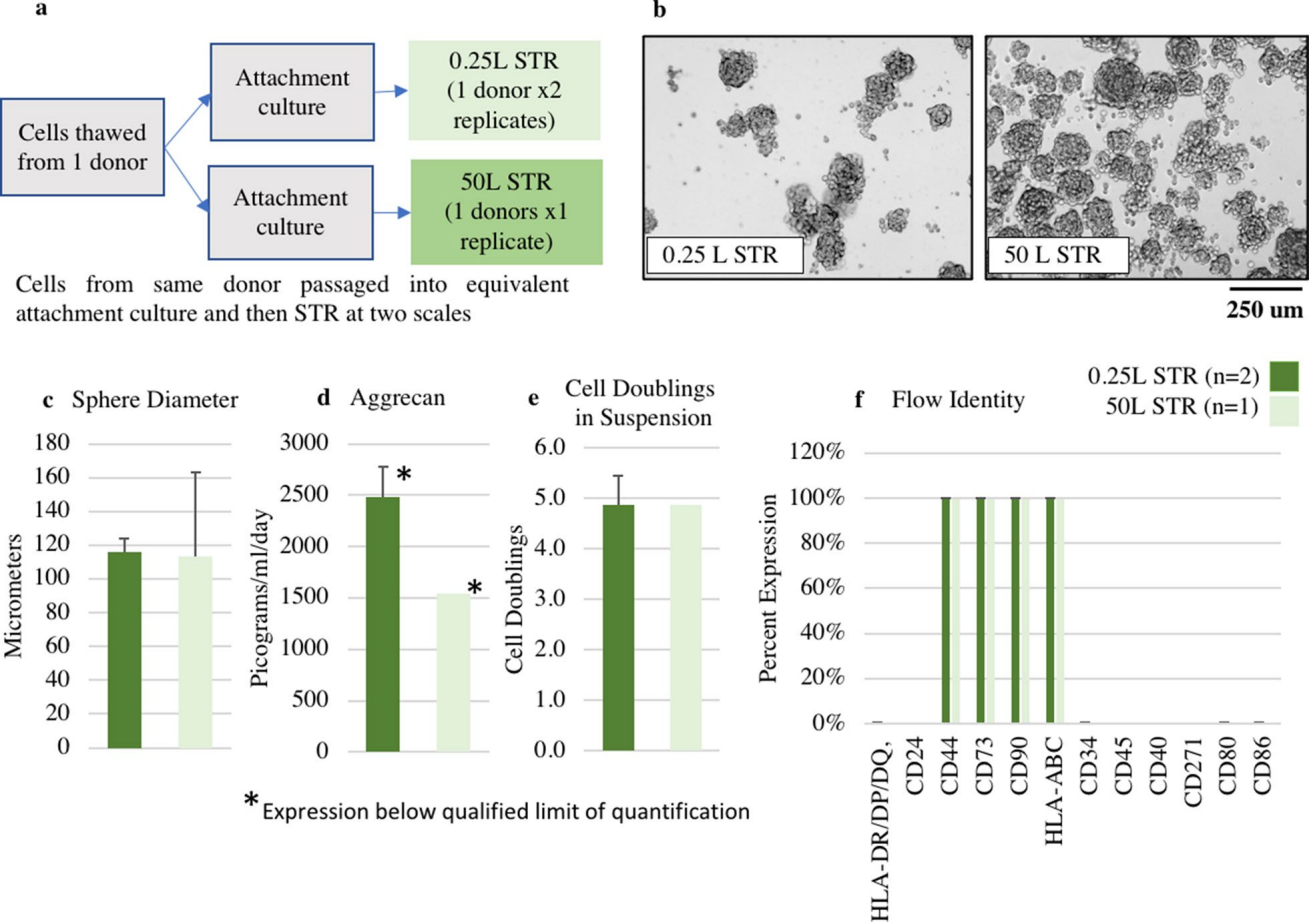
0.25 L versus 50 L STR results. **a** Diagram of our “split stream” growth where 1 donor was grown in both small scale and pilot scale reactors **b** Comparable sphere growth observed in small scale and pilot scale reactors. Sphere size **c** is comparable at harvest. **d** Aggrecan expression is below the qualified limit of detection for both conditions. **e** Cell doublings from day 2 to end of culture across 0.25 L and 50 L STR are comparable. **f** Identity and purity of cells as measured by Flow cytometry is comparable between 0.25 L STR and 50 L STR

Cells were split and grown in 0.25 L and 50 L STRs (Fig. 6a). Maximum shear rate (1/s) was maintained between scales. Spheres formed successfully at both the 0.25 L and 50 L scale (Fig. 6b). There were no statistically significant differences in 50 L and 0.25L STR cell sphere diameter (Fig. 6c). Although the aggrecan value for the 0.25L was higher (2480 picograms/ml/day, SE 210) than the 50L (1540 picograms/ml/day), all aggrecan measurements were below the qualified limit of quantification making interpretation of differences inappropriate (Fig. 6d). Cell doublings between the 0.25L STR (4.86 doublings, SE 0.57) the 50L (4.86 doublings) did not exhibit a statistically significant difference (Fig. 6e). Lastly, flow cytometry found no notable differences in surface markers (Fig. 6f).

## Discussion

Cell sphere production is primarily performed in static, small-scale systems that are unsuitable for large-scale industrialization [1, 2]. The purpose of this work was to transition into a scalable system while maintaining the desirable attributes of the cells established in the static suspension, small-scale systems. To begin our work, we tested our ability to grow cell spheres without a scaffold in wave reactors, Erlenmeyer shake flasks, PBS waterwheels, and STRs. Out of all the modalities tested, the STR was the most promising. With optimization and modeling of hydrodynamic profiles, we were able to understand the impact of maximum shear rate (s^−1^) on our cells and found the appropriate agitation rates necessary to maintain cell attributes in STRs.

Optimizing agitation is a major obstacle in enabling cell sphere culture in STRs [19, 24]. In the STR modality, the run begins by inoculating vessels with a single-cell suspension. Then, these cells expand into progressively larger cell spheres. As the number of cells in each aggregate increases, the mass and buoyancy of the spheres shift. If the agitation setpoint is too low, the cells fall out of solution and attach to the bottom of the vessel. If the agitation is too high, the shear forces prevent cells from forming into spheres and ultimately the cells do not exhibit the desired characteristics. Thus, the goal is to identify an agitation scheme that allows for sphere formation without settling, and this changes as the spheres grow. We found that a ramped agitation scheme enabled sphere formation at the beginning of the run and limited cell settling throughout the run. The use of ramped agitation to keep the Discogenic Cell spheres in suspension differs from the predominate use of magnetic levitation or viscous carriers to grow cell spheres described previously [1, 21, 25].

The optimal conditions identified in our hydrodynamic condition investigations were used for subsequent split stream growth between the original small scale static suspension modality and 0.25L STR suspension methods. Because cell phenotype is impacted by the mechanical, physical and chemical local environment, and the transition from a static culture to a bioreactor includes some significant environmental changes experienced by the cells, it was important to verify that cells grown in STR still resulted in Discogenic Cells with the same final attributes [18, 20, 21, 26]. Both in vitro and in vivo assessments were performed. We found that cells grown in static culture and 0.25L STR exhibited similar doublings, identity, aggrecan production and bioactivity in vivo, measured by increased disc height in rabbit disc degeneration model, as previously seen for Discogenic Cells [12]. To ensure comparability between the pre-clinical and clinical cells, xenograft was successfully used in this model as demonstrated by a lack visual evidence for immune rejection, perhaps due to the avascular nature of the disc making it immune-privileged [27]. Given the xenogeneic nature of this injection, a future study could include tracing the cells to determine the longevity in the disc.

CFD played a key role in aiding the transition into STR and increasing scale by allowing for the efficient optimization of STR parameters. Given the significant expense of running larger volumes of STR, we sought to minimize the number of experiments needed to successfully generate Discogenic Cells. The purpose of maintaining consistent hydrodynamic conditions across modalities and scales is to maintain a similar cell phenotype because hydrodynamic conditions can affect cell differentiation [18, 28–31]. While many hydrodynamic parameters could be used to scale, we found through CFD modeling that Max Shear Rate (1/s) best correlates with aggrecan production and cell doublings for Discogenic Cells, and therefore was most suitable to use for the transition. Aggrecan is believed to be a key element contributing to the potency of this cell therapy, through deposition of tissue commonly found within the disc. Another important finding from CFD that was used in the transition was to control cell volume fraction, which was used to inform what minimum RPM would avoid cell settling. Our modeling suggested that if 3 out of 6 investigated hydrodynamic conditions had been used, instead of Max Shear Rate, the runs would have failed even with ramped agitation. Therefore, the extra modeling effort helped guide the scale-up effort in a useful way to avoid costly, failed runs.

At the 50L scale we were able to demonstrate the feasibility of our scale up strategy generating similar sphere sizes, cell doublings and identity, to the 0.25L scale (Fig. 6). This shows that our use of ramped agitation and maintaining max shear rate between scales has the potential to yield similar results across scales. Cell doublings from this scale up run (4.86 doublings for all conditions) were significantly higher than the previous study where we compared growth in STR (cell doublings = 2.57, SE = 0.39) to the original static suspension method (cell doublings = 2.38, SE = 0.48). However, aggrecan expression was below the qualified limit of quantification at both scales which restricts our ability for meaningful comparison. Because both conditions had low aggrecan values, and we demonstrated successful aggrecan production across 8 other donor cell lines (Fig. 4d), we cannot assume these low values are caused by hydrodynamic conditions. Instead, we hypothesize that cell proliferation may have an inverse correlation to ECM production in our culture. These discrepancies show that future donor, process, and media characterization may be necessary for successful and consistent aggrecan production at any scale. Due to our low sample size of 1 × 50L STR additional studies are a prerequisite for drawing any significant conclusions about scale-up.

While we used aggrecan production to guide our CFD models, other parameters could be used in the future as well. We have demonstrated that other molecules, such as collagen and anti-inflammatory molecules are also relevant to the mode of action of Discogenic Cells in treating lumbar disc degeneration [12, 32]. Further work is needed to evaluate these potency measures in the context of transitioning to a STR and scaling into larger volumes. Ultimately, the data shown for Discogenic Cells grown in a 50 L STR represents a successful proof of concept, and additional studies are needed to further develop optimal process setpoints such as pH, DO, cell, and density which will be further guided by additional potency parameters as well as further understanding of the key mode of action.

Stirred tank bioreactor as a platform technology grants the ability for real time monitoring of cell conditions (pH, DO, etc.) at bench and manufacturing scales, as well as enabling process-controlled scale-up. There is also significant precedence for using STR in approved pharmaceutical products, and equipment can be purchased from many different vendors. For the 50 L STR work, we utilized a Thermo Fisher Scientific TruBio controller that is a DeltaV based automation system for the SUB (STR). It was selected because it is a proven industrial platform for biomanufacturing and supports advanced features important to the long-term success and commercialization of this therapy, such as advanced process analytics, recipe customization, streamlined tech-transfer tools, and meeting cGMP level integrity requirements.

We are currently evaluating the safety and efficacy of Discogenic Cells mixed with a viscous carrier in patients with symptomatic lumbar disc degeneration. The original static suspension process used to produce the cells for the clinical trial must be updated to allow for automation and scale-up, which can suitably meet market demand and compliance for cGMP manufacturing. Generally, cell therapies face manufacturing challenges as products advance beyond clinical trials and enter wide-spread usage. The manufacturing processes for commercial-scale products must be automated, data must be accessible, and larger scale must be successfully utilized. We can utilize well-established bioprocess equipment such as STRs, but must apply novel approaches to scale-up due to the highly sensitive nature of primary human cell lines. CFD was a useful tool in establishing suitable parameters for growing Discogenic Cells in an STR and informing preliminary scale-up parameters. Ultimately, this work advances our efforts to treat painful lumbar disc degeneration and provides a process development framework to utilize in the generation of future cell therapy products.

## Conclusions

- Discogenic cell spheres were successfully grown in a scalable stirred tank bioreactor (STR) modality that can address manufacturing requirements to treat large patient populations.
- Agitation strategy played a key role in successful cell sphere formation. Starting agitation at low speeds allows spheres to form followed by ramping agitation over the course of culture maintains sphere in suspension and controls sphere size.
- Computation Fluid Dynamics modeling of hydrodynamic conditions found max shear rate has significant correlations to aggrecan (*p* = 0.012) and cell doublings (*p* = 0.018).
- Using max shear rate models to tune STR culture aggrecan, cell doublings, and flow identity were maintained between static suspension (*n* = 8) and STR suspension (*n* = 24) culture modalities. Sphere diameter was slightly larger in static suspension (mean diameter = 152 μm, SE 6.8) than STR suspension (mean diameter = 120 μm, SE 6.3)
- Scale up (200-fold) feasibility of the Discogenic cell sphere production process in a stirred tank bioreactor was demonstrated at the 50L scale. By maintaining max shear rate between scales cell sphere production, cell doublings, sphere size, and flow identity were comparable between 0.25L and 50L STRs

## Supplementary Information

**Additional file 1**. Equations and Assumptions used in CFD Modeling

## Data Availability

See Additional file 1

## Abbreviations

CFD: Computational fluid dynamics
STR: Stirred tank reactor
DHI: Disc height index
RPM: Revolutions per minute
DO: Dissolved oxygen
L: Liter
mL: Milliliter
m: Meter
s: Seconds
s^−^^1^: Reciprocal seconds
