# Supplementary material for: Transition from static culture to stirred tank bioreactor for the allogeneic production of therapeutic discogenic cell spheres
Source: Stem Cell Res Ther. 2021 Aug 12;12:455. doi: 10.1186/s13287-021-02525-0 (PMC8359559; doi:10.1186/s13287-021-02525-0)
Supplement: Supplementary file 1 — Additional file 1. Equations and Assumptions used in CFD Modeling [file 13287_2021_2525_MOESM1_ESM.docx]

**Additional File 1 for Transition from Static Culture to Stirred Tank Bioreactors for the Allogeneic Production of Therapeutic Discogenic Cell Spheres**

Daniel Rodriguez-Granrose^1,2^, Jeff Zurawski^1^, Will Heaton^1^, Terry Tandeski^1^, Galina Dulatov^1^, Angelica Adrian Highsmith^1^, Mason Conen^1^, Garrett Clark^1^, Amanda Jones^1^, Hannah Loftus^1^, Cameron LeBaron^1^, Erin Scull^1^, Niloo Farhang,^1^ Isaac Erickson^1^, Justin Bingham^1^, Paula Decaria^4^, Nephi Jones^4^, Kevin T Foley^1,3,5^, Lara Silverman^1,3^

**Affiliations:**

^1^ DiscGenics Inc, Salt Lake City, Utah, USA

^2^ Department of Biochemistry and Molecular Biology, University of Miami, Miami, FL, USA

^3^ Department of Neurosurgery, University of Tennessee Health Science Center, Memphis, Tennessee, USA

^4^ Thermo Fisher Scientific Inc, Logan, Utah, USA

^5^ Semmes-Murphey Clinic, Memphis, Tennessee, USA

**Corresponding Author:**

Daniel Rodriguez-Granrose
[daniel@discgenics.com](mailto:daniel@discgenics.com)

DiscGenics Inc, 5940 Harold Gatty Dr, Salt Lake City, UT 84116

**Additional File 1. Equations, Assumptions, and Conditions Used in CFD Modeling**

The following document outlines the 22 conditions used to link our analytical results to our CFD models as well as the equations and assumptions used in the creation of our CFD. The 22 STR agitation conditions used at 0.25L working volume in Dasbox Mini Reactor systems are shown in **Additional File Table 1.** All changes in impeller revolutions per minute (RPM) were performed on day 2, day 4, and day 5 of culture and maintained until the next indicated RPM.

**Additional File Table 1. RPM by Day in 0.25 L STR reactors**

| Condition Number | Ramped Agitation | RPM at Inoculation (Day 0) | RPM Day 2 | RPM Day 4 | RPM from Day 5 to End of Culture |
| --- | --- | --- | --- | --- | --- |
| 1 | No | 75 | 75 | 75 | 75 |
| 2 | No | 100 | 100 | 100 | 100 |
| 3 | Yes | 100 | 125 | 125 | 125 |
| 4 | No | 125 | 125 | 125 | 125 |
| 5 | Yes | 100 | 100 | 150 | 150 |
| 6 | Yes | 100 | 125 | 150 | 150 |
| 7 | Yes | 100 | 125 | 150 | 150 |
| 8 | Yes | 100 | 125 | 150 | 150 |
| 9 | Yes | 100 | 125 | 150 | 150 |
| 10 | Yes | 100 | 125 | 150 | 150 |
| 11 | Yes | 100 | 125 | 150 | 150 |
| 12 | Yes | 100 | 125 | 150 | 150 |
| 13 | Yes | 100 | 125 | 150 | 150 |
| 14 | No | 150 | 150 | 150 | 150 |
| 15 | Yes | 100 | 100 | 175 | 175 |
| 16 | Yes | 100 | 125 | 150 | 175 |
| 17 | No | 175 | 175 | 175 | 175 |
| 18 | Yes | 100 | 100 | 200 | 200 |
| 19 | Yes | 100 | 125 | 150 | 200 |
| 20 | No | 200 | 200 | 200 | 200 |
| 21 | Yes | 100 | 125 | 150 | 225 |
| 22 | Yes | 100 | 125 | 150 | 250 |

The acronyms and abbreviations used in our CFD calculations and elsewhere are provided in **Additional File Table 2**.

**Additional File Table 2. Acronyms and Abbreviations**

| **Abbreviation** | **Abbreviated Terms in Additional File 1** |
| --- | --- |
| D | impeller diameter (m) |
| kg | Kilograms |
| L | Liter |
| M | moment around impeller (N.m) |
| mL | Milliliter |
| m | Meter |
| N | impeller speed (s^-1^) |
| Po | Power Number |
| P | Power (watts) |
| Re | Reynolds Number |
| RPM | Revolutions per Minute |
| s | Seconds |
| s^-1^ | Reciprocal seconds |
| V | Volume (m^3^) |
| ρ | fluid density (kg.m^-3^) |
| μ | liquid viscosity (kg/m/s) |
| γ | average shear rate (s^-1^) |
| γ_imp,tur_ | impeller averaged turbulent average shear rate (s^-1^) |

The most critical assumption is that the Reynolds Number (a unitless coefficient which describes the flow characteristics) is in the turbulent flow region i.e. Reynolds Number > 2500 (Turbulent) [33]. Reynold number was calculated using the equation:

The power input can be calculated in CFD via:

𝑃 = 𝜋NM

The impeller power number, Po (-) can then be calculated:

The shear rate calculations specifically relate to the energy dissipation in the vessel. As the energy introduced by the impeller is dissipated, this energy is consumed by the nearby fluid and other particles (specifically in this case, proteins) which can be negatively impacted by a high rate of energy dissipation (i.e. high shear). The shear rate calculations are computed as follows:

Vessel averaged turbulent shear rate:

Impeller averaged turbulent shear rate:

Where:

.
